# Tenosynovial Giant Cell Tumor of Foot in a Patient with Radioiodine-Refractory Thyroid Cancer: Imaging Findings on FDG-PET/CT, MRI, and Radioiodine Scan

**DOI:** 10.1055/s-0042-1758804

**Published:** 2022-12-20

**Authors:** Sarvesh Loharkar, Sandip Basu

**Affiliations:** 1 Radiation Medicine Centre, Bhabha Atomic Research Centre, Tata Memorial Hospital Annexe, Parel, Mumbai, Maharashtra, India; 2 Homi Bhabha National Institute, Mumbai, Maharashtra, India

**Keywords:** benign tenosynovial giant cell tumor, ^18^ F-FDG-PET/CT, benign bone tumor, radioiodine refractory thyroid cancer, thyroglobulin elevated negative iodine scintigraphy, differentiated thyroid carcinoma

## Abstract

We herein illustrate a case of benign tenosynovial giant cell tumor, which was incidentally detected as FDG-avid lesion on PET/CT in a patient with radioiodine refractory thyroid cancer, with predominantly non-iodine concentrating disease. The lesion was followed up clinically and with local MRI annually for subsequent 3 years. The utility of hybrid PET-CT imaging, the non-iodine concentration of the tumor along with clinical knowledge, and findings on other imaging and pathological modalities in answering and diagnosing incidental benign musculoskeletal tumors in a patient with known thyroid malignancy are presented here.

## Introduction

Patients with radioactive-iodine refractory (RAIR) thyroid cancer and thyroglobulin elevated negative iodine scintigraphy (TENIS) are considered as therapeutic challenges and possess guarded prognosis compared to their DTC counterparts. FDG-PET/CT has become a pivotal imaging tool in these cases, its sensitivity and is positively correlated with serum thyroglobulin (Tg) levels and plays a prognostic indicator in this patient subset. 1 2 The false-positive uptake of FDG in multiple other neoplastic as well as non-neoplastic forms warrants careful interpretation, which may also be observed in benign bone neoplasms. Additionally, DTCs have been incidentally detected with other malignancies and vice versa, the most common being breast cancers. 3 These facts warrant clinical correlation and cautiousness while labeling FDG uptake as part of metastatic disease involvement in such cases. Here we discuss a case of RAIR thyroid cancer, which was imaged using an FDG-PET/CT scan and found to have FDG-avid benign tenosynovial giant cell tumor (BTSGCT) of the foot.

## Case Report

**Fig. 1:**
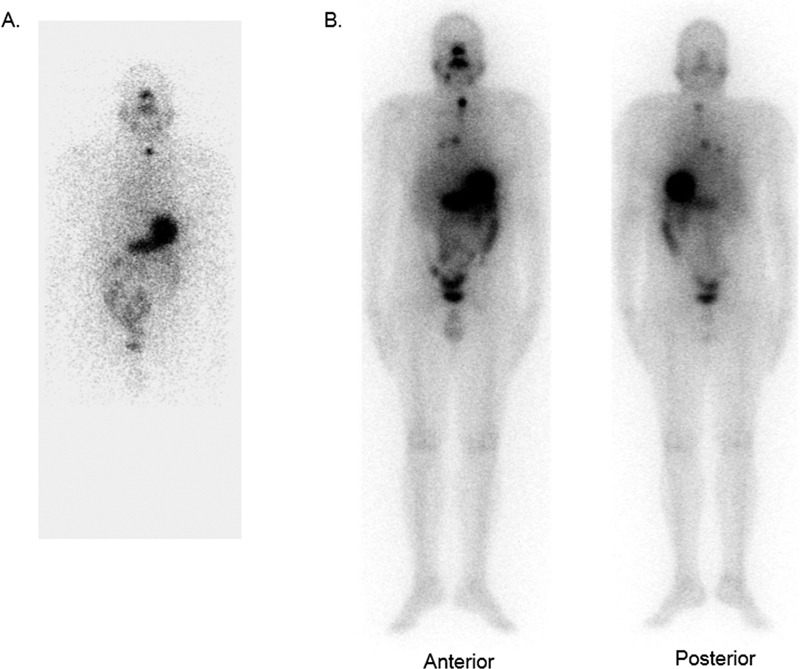
1-mCi radioactive iodine planar scan ( **A** ) showing tracer uptake in the midline neck region (thyroid bed), and ( **B** ) post-radioiodine therapy scan showing focal tracer concentration in the neck, chest, and low-grade diffuse chest uptake.

**Fig. 2:**
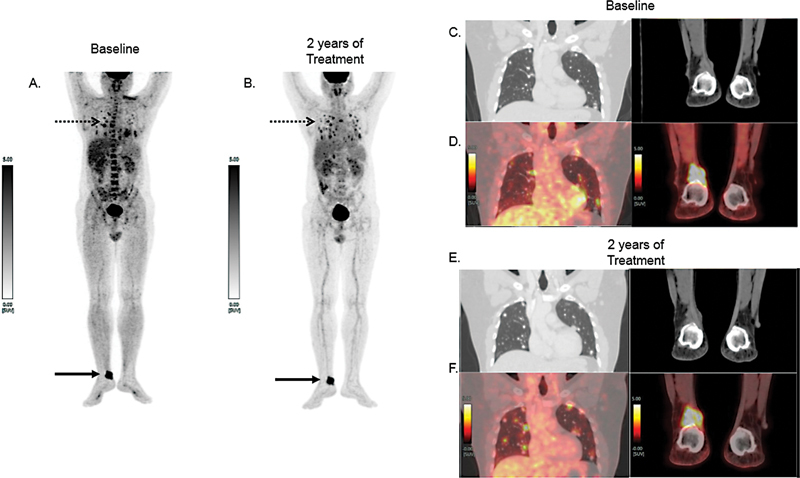
FDG-PET/CT at baseline ( **A** ) and 2 years of treatment ( **B** ) showing FDG concentrating tiny multiple bilateral lung nodules, increased in number ( *dotted arrow* ). FDG avid subcutaneous soft tissue density lesion at the right ankle ( *solid arrow* ). On the right panel, the lung and foot lesions are demonstrated with their enlarged coronal views, at pre-treatment and at 2 years following sorafenib therapy. Of these, the left column shows CT chest images (lung window) coronal section ( **C** ) and ( **E** ); PET/CT coronal chest fused images ( **D** ) and ( **F** ). Right column CT images coronal section at foot level ( **C** ) and ( **E** ); and corresponding fused PET/CT images ( **D** ) and ( **F** ) at baseline and 2 years of treatment are also illustrated.

A 50-year-old male patient, diagnosed with differentiated papillary thyroid carcinoma (classical type), with extensive capsular invasion and extra-thyroidal extension and bilateral neck lymph node, and lung metastasis (on preoperative evaluation with regional CT scan), had undergone total thyroidectomy with bilateral central compartment clearance with bilateral modified neck dissection. The strap muscles also showed metastatic deposits, and multiple resected nodes were positive for metastases and showed extra-nodal extension. His RAI scan ( Fig. 1A ) showed only a small focus of uptake in the neck, following which he received high-dose RAI therapy with the traditional protocol of TSH elevation following thyroid hormone withdrawal (the pre-treatment TSH level was 36 µIU/mL). He received a therapeutic dose of 7,326 MBq of ^131^ I as per the institutional protocol, and the post-RAI therapy scan ( Fig. 1B ) showed neck uptake and faint bilateral chest uptake. The TSH stimulated serum Tg was 432.21 ng/mL before therapy and he was considered for whole-body FDG-PET/CT, especially in view of that preoperatively noted lesions were not well visualized on RAI scan. FDG-PET/CT showed multiple nodular lesions in bilateral lungs, many of which showed intense FDG concentration ( Fig. 2A , left column of C and D). One FDG avid subcutaneous soft tissue lesion was noted in the right ankle region, just superior to the calcaneus measuring 3.4 × 3.0 cm with an SUVmax value of 12.07; subtle cortical erosion of the underlying calcaneus bone was also noted ( Fig. 2A , right columns of C and D).

**Fig. 3:**
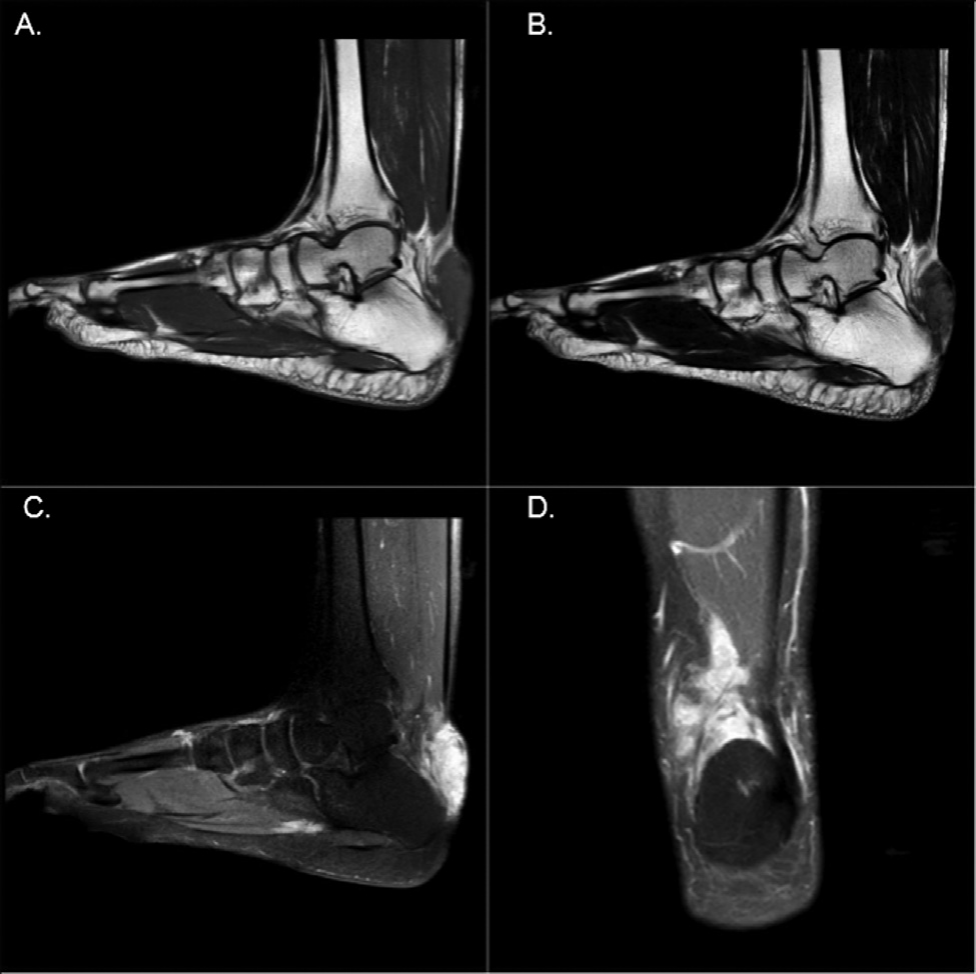
MR image of the right foot and ankle showing ( **A** ) T1-weighted sagittal section image demonstrating T1 isointense soft tissue lesion in the subcutaneous region of the posterior aspect of the foot superior to the calcaneum, encasing the Achilles tendon without infiltration. ( **B** ) T2-weighted sagittal section image showing intermediate intensity of the lesion with no obvious erosions; ( **C** and **D** ) dynamic post (gadolinium) contrast T1-weighted images showing homogenous post-contrast enhancement in the lesion.

The patient was asymptomatic except for some local swelling; on local examination, there was soft to firm swelling without skin fixity over the Achilles tendon of the right ankle, which, according to the patient, was stable over last 18 months and was non-tender. A local ultrasonographic examination done showed a heterogeneous soft tissue lesion in the subcutaneous plane superficial to the Achilles tendon in the dorsal aspect of the right ankle measuring 2.9 × 2 × 0.6 cm. The lesion was further elucidated with a local MRI done after RAI therapy ( Fig. 3 ). This revealed irregular soft tissue lesion in subcutaneous region of the posterior aspect of foot, just superior to the calcaneus, encasing the tendon Achilles without infiltration. It was seen abutting the cortex of the calcaneum with no appreciable erosion. MR characteristics of lesion were isointense on T1, intermediate on T2, and hyperintense on STIR, with minimal restricted diffusion and homogenous post-contrast enhancement. Though these MRI characteristics were not typical for metastasis, considering the diagnosis of malignancy in the patient, FNAC was planned. The FNAC showed scattered osteoclastic giant cells, numerous histiocytes, and lymphocytes and was labeled as a BGCTS. Retrospectively, post-therapy whole body RAI scans ( Fig. 1B ) also did not reveal any radioiodine concentration in this lesion. This lesion was followed up clinically and local MRI every 12 months.

In his further course, he developed non-radioiodine concentrating disease and elevated serum Tg with FDG concentrating multiple bilateral lung nodules and started on tyrosine kinase inhibitor (lenvatinib) therapy. At nearly 2 years down the line, follow-up FDG-PET/CT study ( Fig. 2B, E, F ) demonstrated some increase in his metabolically active lung metastatic lung lesions but almost stable metabolically active right-sided ankle lesion.

## Discussion

BTSGCT is one of the rare benign bone tumors usually affecting adults and are limited to specific joint areas. A few case reports have reported this to be FDG avid, and FDG-PET/CT along with MRI is useful in evaluating its local extent and the response to CSF1-targeting therapies. Nervo et al recently performed an extensive study on bone disease in thyroid cancer and suggested the rare possibility of benign bone tumors in such setting and the utility of MRI, especially the DWI sequence, in their diagnosis. 4 The same is evidenced in the present case, differentiating it from the metastatic spread, and confirmed on minimally invasive FNAC technique and follow-up scans. To the best of our knowledge, this is the first such case reported in a patient having metastatic DTC. Pallas et al have reported similar such FDG avid BGCTS tumor mimicking melanoma metastases. 5

In the literature, there are descriptions of benign tumors concentrating radioiodine. This includes (1) parotid tumors such as Warthin's tumor or oncocytoma, (2) meningioma, (3) cavernous angioma, (4) cystic mesothelioma, (5) hepatic hemangioma, (6) breast fibroadenoma, (7) uterine myoma, (8) struma cordis and struma ovarii along with other types of ovarian tumors and teratoma. These findings have been related to NIS-dependent uptake or other mechanisms 6 and potentially may cause false-positive results in routine RAI scans. There are other reports of incidental findings of various types of BTSGCT showing uptake on FDG-PET/CT acquired for other indications, mostly in oncology patients. This case uniqueness was in the incidence of this benign tumor in a patient with thyroid cancer imaged and treated with radioactive iodine. Despite the known uptake of RAI in certain other benign tumors, this lesion was cold on the RAI scan and hence underscored the non-radioiodine concentrating property of BTSGCT. A confounding scenario in this patient was high serum Tg values, which could be explained by FDG-avid extensive bilateral pulmonary metastases.
